# Activation of α7 Nicotinic Acetylcholine Receptor Decreases On-site Mortality in Crush Syndrome through Insulin Signaling-Na/K-ATPase Pathway

**DOI:** 10.3389/fphar.2016.00079

**Published:** 2016-03-29

**Authors:** Bo-Shi Fan, En-Hui Zhang, Miao Wu, Jin-Min Guo, Ding-Feng Su, Xia Liu, Jian-Guang Yu

**Affiliations:** ^1^Department of Pharmacology, Second Military Medical UniversityShanghai, China; ^2^The 406th Hospital of Chinese People’s Liberation ArmyDalian, China; ^3^Jinan Military General HospitalJinan, China

**Keywords:** *α*7 nicotinic acetylcholine receptor, crush syndrome, mortality, hyperkalemia, insulin sensitivity, Na/K-ATPase

## Abstract

On-site mortality in crush syndrome remains high due to lack of effective drugs based on definite diagnosis. Anisodamine (Ani) is widely used in China for treatment of shock, and activation of α7 nicotinic acetylcholine receptor (α7nAChR) mediates such antishock effect. The present work was designed to test whether activation of α7nAChR with Ani decreased mortality in crush syndrome shortly after decompression. Sprague-Dawley rats and C57BL/6 mice with crush syndrome were injected with Ani (20 mg/kg and 28 mg/kg respectively, i.p.) 30 min before decompression. Survival time, serum potassium, insulin, and glucose levels were observed shortly after decompression. Involvement of α7nAChR was verified with methyllycaconitine (selective α7nAChR antagonist) and PNU282987 (selective α7nAChR agonist), or in α7nAChR knockout mice. Effect of Ani was also appraised in C2C12 myotubes. Ani reduced mortality and serum potassium and enhanced insulin sensitivity shortly after decompression in animals with crush syndrome, and PNU282987 exerted similar effects. Such effects were counteracted by methyllycaconitine or in α7nAChR knockout mice. Mortality and serum potassium in rats with hyperkalemia were also reduced by Ani. Phosphorylation of Na/K-ATPase was enhanced by Ani in C2C12 myotubes. Inhibition of tyrosine kinase on insulin receptor, phosphoinositide 3-kinase, mammalian target of rapamycin, signal transducer and activator of transcription 3, and Na/K-ATPase counteracted the effect of Ani on extracellular potassium. These findings demonstrated that activation of α7nAChR could decrease on-site mortality in crush syndrome, at least in part based on the decline of serum potassium through insulin signaling-Na/K-ATPase pathway.

## Introduction

Crush injury is defined as compression of the extremities or other parts of the body, resulting in muscle swelling and/or neurological disturbances in the affected areas of the body (Oda et al., 1997). Based on crush injury, crush syndrome is characterized by systemic symptoms due to rhabdomyolysis including acute kidney injury, hypovolemic shock, and metabolic disorders (e.g., hyperkalemia), etc. (Better, 1997; Oda et al., 1997; Bywaters and Beall, 1998; Slater and Mullins, 1998; Sever et al., 2015), which appears to be common in disasters, wars, and acts of terrorism. Crush syndrome in violent earthquakes, with an incidence about 2∼15%, is the most frequent cause of death, apart from trauma (Ukai, 1997). Up to 20% crush victims died of cardiac arrest caused by hyperkalemia or hypovolemic shock in a short time (Ashkenazi et al., 2005). However, safe and effective drugs to reduce on-site mortality in crush syndrome are clinically vacant, due to lack of definite diagnosis (Sever and Vanholder, 2013).

Anisodamine (Ani) is a belladonna alkaloid isolated from the Chinese medicinal herb *Scopolia tangutica* Maxim of the Solanaceae family indigenous to Tibet. It is used clinically to improve blood flow in circulatory disorders such as septic shock and disseminated intravascular coagulation. Our previous studies found that activation of α7 nicotinic acetylcholine (ACh) receptor (α7nAChR) is involved in the antishock effect of Ani (Liu et al., 2009). We speculated that Ani could reduce mortality in crush syndrome shortly after decompression through activation of α7nAChR. A series of experiments on crush syndrome in rats and α7nAChR knockout (α7^-/-^) mice were designed to test this hypothesis. As known, insulin can promote entrance of potassium into cells. The effect of insulin on serum potassium has been attributed to activation of Na/K-ATPase (Chibalin et al., 2001; Al-Khalili et al., 2004; Benziane and Chibalin, 2008). Chronic exposure to nicotine could enhance insulin sensitivity via α7nAChR (Wang et al., 2011; Xu et al., 2012). We speculated that activation of α7nAChR with Ani could decrease serum potassium through elevation of insulin sensitivity.

The present work was designed to test the effectiveness of activating α7nAChR on reduction of on-site mortality in crush syndrome, and to demonstrate the signaling pathway involved. Here, we show for the first time that activation of α7nAChR could decrease on-site mortality in crush syndrome. Decline of serum potassium by activating α7nAChR is intimately linked to insulin signaling-Na/K-ATPase pathway.

## Materials and Methods

### Animals and Reagents

Sprague-Dawley rats (230∼270 g) and C57BL/6 mice (25∼30 g) were purchased from Sino-British SIPPR/BK Laboratory Animals (Shanghai, China). α7^-/-^ mice were generated and genotyped by PCR analysis as described previously (Liu et al., 2009). Animals were housed at 22°C under a 12/12 light schedule (on: 08:00), with free access to tap water and standard rat chow. All experimental procedures were in accordance with institutional animal care guidelines and approved by ethics committee of Second Military Medical University. Ani (Ani hydrochloride: C_17_H_24_NO_4_) was purchased from Fu-Ma Chemical and Engineering Company (Hangzhou, China). Mecamylamine hydrochloride, methyllycaconitine (MLA) citrate, hexamethonium chloride, ACh chloride, nicotine, PNU282987 (PNU), static, ouabain octahydrate, potassium chloride, and antibody against α7nAChR were purchased from Sigma–Aldrich (St. Louis, MO, USA). HNMPA-(AM)3 and antibody against p-Na/K-ATPase were purchased from Santa Cruz Biotechnology (Dallas, TX, USA). Antibody against insulin receptor, Alexa-488-labeled and Cy3-labeled second antibodies, and DAPI were purchased from Abcam (Cambridge, MA, USA). LY 294002 and rapamycin were purchased from Merck Millipore (Darmstadt, Germany).

### Preparation of Crush Syndrome Models

Rats were anesthetized with a combination of ketamine (10 mg/kg, i.p.) and diazepam (0.1 mg/kg, i.p.) after an overnight fast, while mice were anesthetized with a combination of ketamine (15 mg/kg, i.p.) and diazepam (0.15 mg/kg, i.p.) after 6-h fast. The animals were fixed in prone position, with hind limbs (4.5 and 2 cm from the ankles up for rats and mice respectively) compressed by 20 kg weights for 5 h. The mortality was 70–80% at 24 h after decompression.

### Cell Culture and Differentiation

Mouse C2C12 myoblasts were kindly provided by Stem Cell Bank, Chinese Academy of Sciences and cultured with DMEM supplemented with 10% FBS, 2 mmol/L glutamate, 15 mmol/L HEPES, 100 IU/ml penicillin, and 100 mg/ml streptomycin in 95% O_2_ and 5% CO_2_. To obtain fully differentiated myotubes, FBS was removed from cell culture at 70% confluence, and cells were incubated in a medium containing 2% horse serum for 4 additional days.

### Serum Biochemical Assays

Serum creatine kinase (CK), CK isoenzyme-MB (CK-MB), blood urea nitrogen (BUN), serum creatinine (Scr), K^+^, Na^+^, Cl^-^, and glucose levels in rats or mice and extracellular K^+^ and glucose levels in cultured C2C12 myoblasts were measured with an autoanalyzer (Beckman Autoanalyzer; Beckman Instruments, Fullerton, CA, USA). Rats and mice serum insulin levels were determined by ELISA according to the manufacturer’s instructions (Shibayagi, Gunma, Japan). The homeostasis model assessment of insulin resistance (HOMA-IR) index was calculated according to the following formula: HOMA-IR = fasting serum insulin (mIU/L) × fasting serum glucose (mmol/L)/22.5. The quantitative insulin sensitivity check index (QUICKI) was calculated using the original formula as the inverse log sum of fasting serum insulin in mIU/L and fasting serum glucose in mg/dl. QUICKI = 1/[log(fasting serum glucose) + log(fasting serum insulin)].

### Immunofluorescence

C2C12 cell cultures were carried out directly on glass coverslips overnight at 37°C. Cells were rinsed with PBS, fixed with 4 % paraformaldehyde for 15 min at room temperature, and then rinsed with PBS twice before blocked with 5% BSA in PBS with Tween 20 for 30 min. Cells were incubated with primary antibodies against α7nAChR, insulin receptor, or p-Na/K-ATPase for 2 h at 37°C. Subsequently, cells were washed three times for 15 min and incubated with Alexa-488 or Cy3-labeled second antibodies for 1 h at 37°C. Nuclei were labeled with DAPI for 5 min. After rinsed, glass coverslips were mounted on glass slides and the cells were visualized under confocal laser scanning microscope (Park et al., 2014; Tan et al., 2014). Eight independent fields were acquired from each coverslip. Measurements were performed as follows: integrated optical density (IOD) and total fluorescent area (TFA) in each image was recorded using the Image-Pro Plus 6.0 software (Media Cybernetics, Bethesda, MD, USA). Mean optical density (MOD) = IOD/TFA.

### Experimental Protocols

#### Experiment 1: Effect of Ani on Mortality and Serum Biochemicals in Rats with Crush Syndrome

Rats were randomly divided into four groups (*n* = 20 per group): (1) normal: rats received normal saline (i.p.); (2) control: rats received normal saline (i.p.); (3) Ani-pre: rats received Ani (20 mg/kg, i.p.) at 30 min before decompression; (4) Ani-post: rats received Ani (20 mg/kg, i.p.) at 1 h after decompression. Crush syndrome models were established in groups 2–4. Blood samples of all the rats were collected from orbital venous sinus at 6 h after decompression, and serum CK, CK-MB, BUN, Scr, K^+^, Na^+^, Cl^-^, insulin, and glucose levels were measured. Survival time was monitored for 24 h after decompression.

#### Experiment 2: Involvement of α7nAChR in the Effect of Ani on Mortality, Serum K^+^, and Insulin Sensitivity in Mice with Crush Syndrome

C57BL/6 mice were randomly divided into five groups (*n* = 15 per group): (1) control: mice received normal saline (i.p.); (2) Ani (28 mg/kg); (3) MLA (10 mg/kg); (4) Ani (28 mg/kg) + MLA (10 mg/kg); (5) PNU (4 mg/kg). Crush syndrome models were established. Ani or PNU was given i.p. at 30 min before decompression, and MLA was given i.p. 30 min earlier. Survival time was monitored for 24 h after decompression. Crush syndrome models were established in another five groups of C57BL/6 mice (*n* = 6 per group) as listed above. Blood samples were collected from vena cava at 6 h after decompression, and serum K^+^, insulin, and glucose levels were measured, as well as in a “normal” group. In another set of experiments, normal saline or Ani (28 mg/kg) was given i.p. to α7^-/-^ mice and wild-type (WT) controls at 30 min before decompression. Survival time was monitored for 24 h after decompression (*n* = 18 per group), and serum K^+^, insulin, and glucose levels were measured at 6 h after decompression (*n* = 6 per group).

#### Experiment 3: Effect of Ani on Mortality and Serum K^+^ in Rats with Hyperkalemia

Rats were randomly divided into two groups (*n* = 18 per group), receiving normal saline (i.p.) or Ani (20 mg/kg, i.p.), and KCl (750 mg/kg, i.p.) was given 30 min later. Blood samples were collected from orbital venous sinus at 30 min after KCl injection and from heart for those just died, and serum K^+^ level was measured. Survival time was monitored for 24 h after KCl injection.

#### Experiment 4: Involvement of α7nAChR in the Effect of Ani on Extracellular K^+^ *In Vitro*

Myotubes were pretreated for 30 min with Ani (500 μg/ml), ACh (100 μmol/L), Ani (500 μg/ml) + ACh (100 μmol/L), nicotine (0.01–10 μmol/L), or PNU (0.01–10 μmol/L) prior to exposure to KCl (5 mmol/L), and extracellular K^+^ and glucose levels were measured 6 h later. In another set of experiments, myotubes were pretreated for 1 h with mecamylamine (10 μmol/L), MLA (10 μmol/L), or hexamethonium (10 μmol/L), and then for 30 min with Ani (500 μg/ml) + ACh (100 μmol/L) or nicotine (1 μmol/L) prior to exposure to KCl (5 mmol/L), and extracellular K^+^ and glucose levels were measured 6 h later.

#### Experiment 5: Influence of HNMPA-(AM)3 on the Effect of Ani on Na/K-ATPase Phosphorylation *In Vitro*

In these experiments, myotubes were divided into five groups: (1) normal; (2) control; (3) Ani (500 μg/ml) + ACh (100 μmol/L); (4) HNMPA-(AM)3 (200 μmol/L); (5) Ani (500 μg/ml) + ACh (100 μmol/L) + HNMPA-(AM)3 (200 μmol/L). Myotubes were pretreated for 1 h with HNMPA-(AM)3, and then for 30 min with Ani + ACh prior to exposure to KCl (5 mmol/L). Phosphorylation of Na/K-ATPase was then detected with immunofluorescence staining and confocal microscopy. Expression of α7nAChR and insulin receptor was also examined in normal C2C12-differentiated myotubes with immunofluorescence.

#### Experiment 6: Influences of Insulin Signaling Blocking on the Effect of Ani on Extracellular K^+^ *In Vitro*

Myotubes were pretreated for 1 h with HNMPA-(AM)3 (200 μmol/L), LY 294002 (10 μmol/L), rapamycin (200 nmol/L), stattic (10 μmol/L), or ouabain (2 mmol/L), and then for 30 min with Ani (500 μg/ml) + ACh (100 μmol/L) prior to exposure to KCl (5 mmol/L), and extracellular K^+^ and glucose levels were measured 6 h later.

### Statistical Analysis

Data are expressed as mean ± SD. For experiments involving only two groups, data were analyzed with *t*-test or *t*’-test. For experiments involving more than two groups, data were analyzed with ANOVA followed by Bonferroni test or Games-Howell test. Kaplan–Meier analysis, followed by a log-rank test, was used for survival time analysis. *P* < 0.05 was considered statistically significant. Survival rate between two groups were analyzed with Fisher’s exact test. The size of experimental groups was determined according to statistical requirement, and power analysis was performed. Analyses were performed using SPSS 21.0 (SPSS, Inc., Chicago, IL, USA) and PASS 11.0 (NCSS, LLC, Kaysville, UT, USA).

## Results

### Effect of Ani on Mortality and Serum Biochemicals in Rats with Crush Syndrome

**FIGURE 1:**
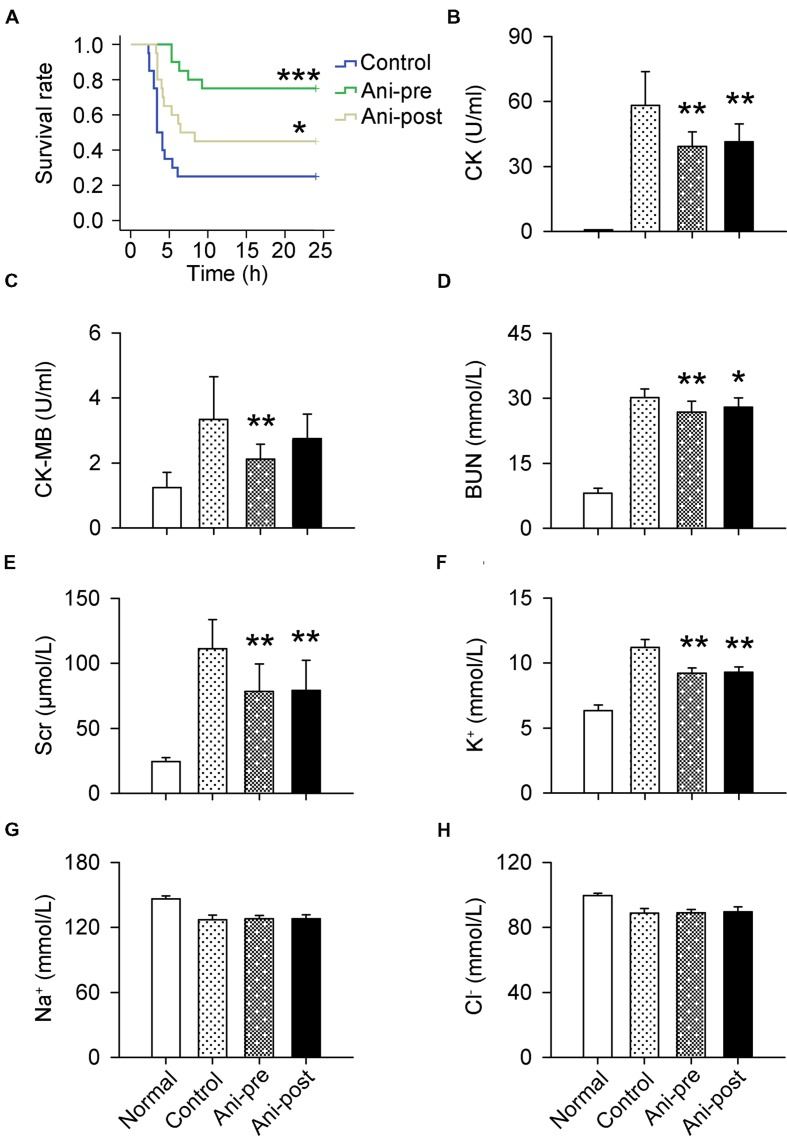
**Effect of Ani on mortality and serum biochemicals in rats with crush syndrome.** Ani (20 mg/kg, i.p.) was administrated 30 min before (Ani-pre) or 1 h after (Ani-post) decompression. Blood samples were collected at 6 h after decompression. Ani prolonged survival time after decompression **(A)**. Serum CK, CK-MB, BUN, Scr, and K^+^ levels were significantly reduced by Ani **(B–F)**. Ani had no effect on serum Na^+^ and Cl^-^ levels **(G,H)**. *N* = 20 per group. ^∗^*P* < 0.05, ^∗∗^*P* < 0.01, ^∗∗∗^*P* < 0.001 vs. control. Power = 0.90, 1.00, 1.00, 1.00, 1.00, 1.00, 0.11, and 0.13 for **(A–H)** respectively.

The mortality rate within 24 h was much lower in Ani-pre and Ani-post groups compared with that in control group (25 and 55% vs. 75%). Kaplan–Meier analysis revealed significantly longer survival time in Ani-pre group (log-rank testing χ^2^ = 14.35, *P* = 0.00015) and Ani-post group (log-rank testing χ^2^ = 3.70, *P* = 0.049, **Figure 1A**) compared with that in control group. Serum CK, CK-MB, BUN, Scr, and K^+^ levels at 6 h after decompression were significantly lower in Ani treated rats, especially in Ani-pre group (*P* < 0.01, **Figures 1B–F**) compared with that in control group. Treatment with Ani had no effect on serum Na^+^ and Cl^-^ levels (**Figures 1G,H**).

### Ani Decreases Mortality and Serum K^+^ in Mice with Crush Syndrome through Activating α7nAChR

**FIGURE 2:**
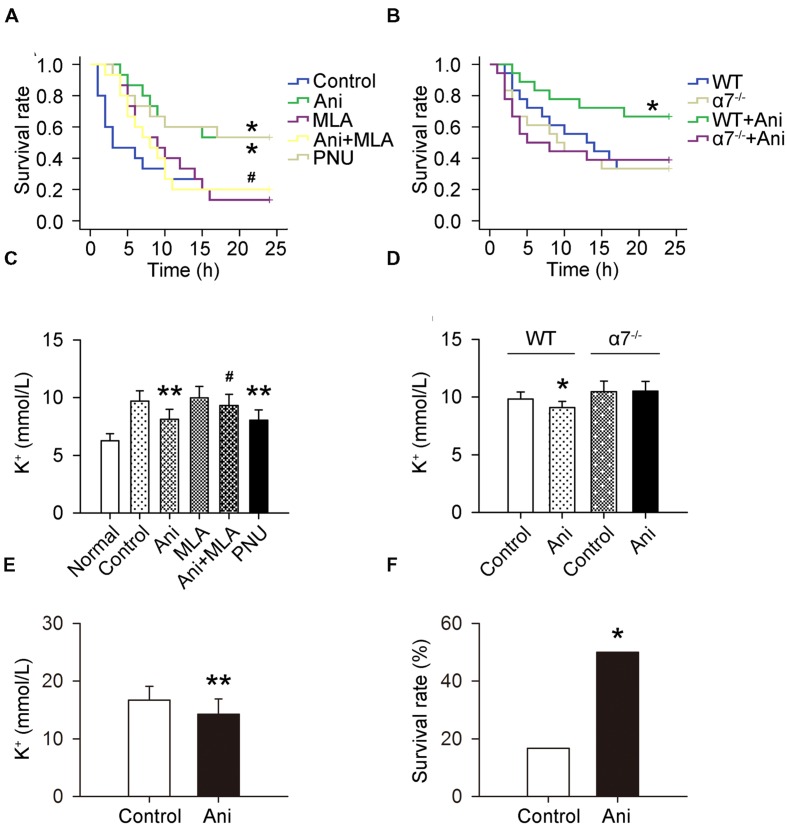
**Involvement of α7nAChR in the effect of Ani on mortality and serum K^+^ in mice with crush syndrome and effect of Ani on mortality and serum K^+^ in rats with hyperkalemia.** Ani (28 mg/kg) or PNU (4 mg/kg) was administrated i.p. at 30 min before decompression in mice with crush syndrome, and MLA (10 mg/kg, i.p.) was given 30 min earlier. Blood samples were collected at 6 h after decompression. Ani and PNU prolonged survival time (*n* = 15 per group) and decreased serum K^+^ level (*n* = 6 per group) in C57BL/6 mice after decompression, and MLA significantly counteracted such beneficial effects of Ani **(A,C)**. Ani prolonged survival time (*n* = 18 per group) and decreased serum K^+^ level (*n* = 6 per group) in WT mice for α7nAChR after decompression, which was abolished in α7^-/-^ mice **(B,D)**. Ani (20 mg/kg, i.p.) was administrated 30 min before injection of KCl (750 mg/kg, i.p.), and blood samples were collected 30 min after KCl injection. Ani prolonged survival time and decreased serum K^+^ level in rats with hyperkalemia (*n* = 18 per group, **E,F**). ^∗^*P* < 0.05, ^∗∗^*P* < 0.01 vs. control. ^#^*P* < 0.05 vs. Ani. Power = 0.60, 0.62, 0.97, 1.00, 0.84, and 0.70 for **(A–F)** respectively.

The mortality rate within 24 h was much lower in Ani and PNU groups compared with that in control group (46.7 and 46.7% vs. 80%), and much higher in Ani + MLA group compared with that in Ani group (80.0% vs. 46.7%). Kaplan–Meier analysis revealed significantly longer survival time in Ani group (log-rank testing χ^2^ = 5.80, *P* = 0.016) and PNU group (log-rank testing χ^2^ = 5.37, *P* = 0.020) compared with that in control group, and significantly shorter survival time in Ani + MLA group (log-rank testing χ^2^ = 4.06, *P* = 0.044, **Figure 2A**) compared with that in Ani group. Serum K^+^ level at 6 h after decompression was significantly lower in Ani (-16.4%, *P* < 0.01) or PNU (-17.2%, *P* < 0.01) treated rats compared with that in control group, and significantly higher in Ani + MLA group compared with that in Ani group(+15.0%, *P* < 0.05, **Figure 2C**). Ani significantly decreased the mortality rate (-50.0%), prolonged survival time (log-rank testing χ^2^ = 3.88, *P* = 0.049, **Figure 2B**), and decreased serum K^+^ level (-7.4%, *P* < 0.05, **Figure 2D**) in WT mice, but had no effect in α7^-/-^ mice.

### Ani Decreases Mortality and Serum K^+^ in Rats with Hyperkalemia

Serum K^+^ was much lower in Ani group compared with that in control group (-14.7%, *P* < 0.01, **Figure 2E**), and the mortality rate within 24 h was also much lower in Ani-treated rats compared with that in control rats (50.0% vs. 83.3%, *P* < 0.05, **Figure 2F**). All death occurred within 30 min after KCl injection.

### Ani Increases Insulin Sensitivity in Animals with Crush Syndrome through Activating α7nAChR

**FIGURE 3:**
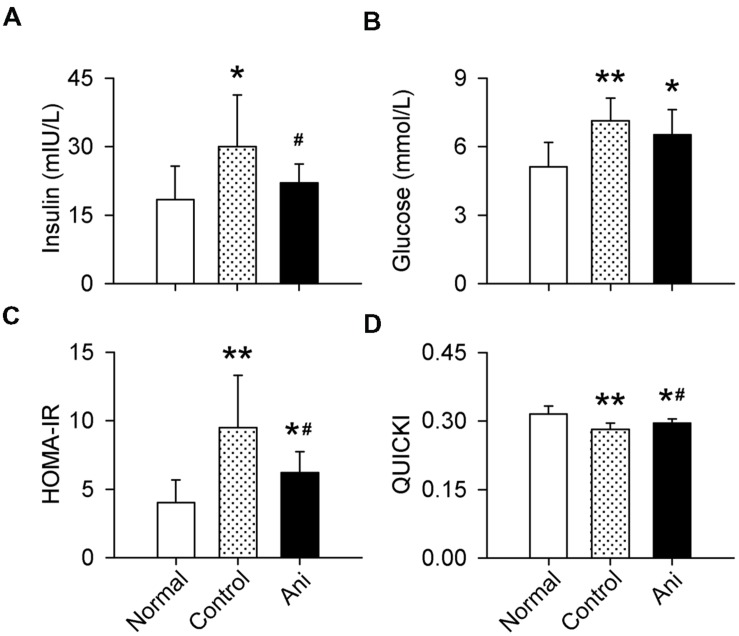
**Effect of Ani on insulin sensitivity in rats with crush syndrome.** Ani (20 mg/kg, i.p.) was administrated 30 min before decompression. Blood samples were collected at 6 h after decompression. Ani decreased serum insulin **(A)**, but had no significant effect on glucose **(B)** in rats after decompression. Ani improved insulin sensitivity, reflected by decreased HOMA-IR index **(C)** and increased QUICKI **(D)**. *N* = 20 per group. ^∗^*P* < 0.05, ^∗∗^*P* < 0.01 vs. normal. ^#^*P* < 0.05 vs. control. Power = 1.00 for **(A–D)**.

**FIGURE 4:**
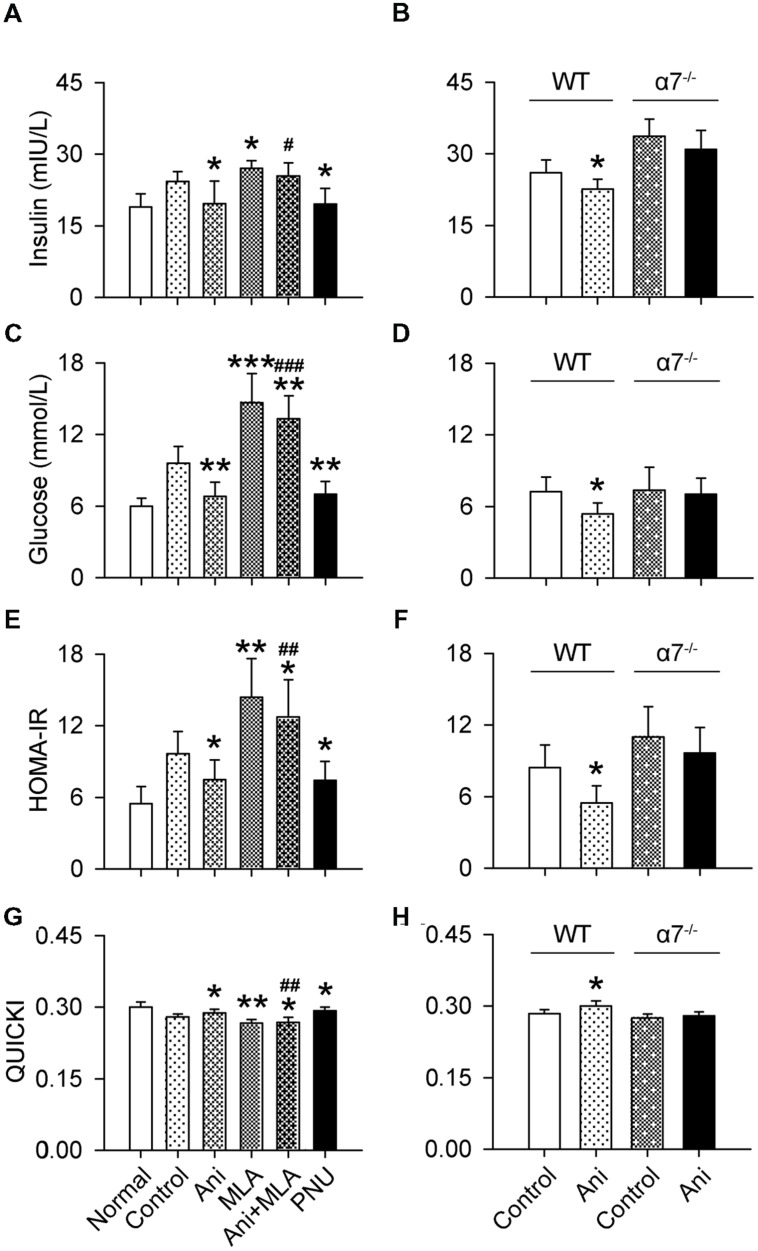
**Involvement of α7nAChR in the effect of Ani on insulin sensitivity in mice with crush syndrome.** Ani (28 mg/kg) or PNU (4 mg/kg) was administrated i.p. at 30 min before decompression in mice with crush syndrome, and MLA (10 mg/kg, i.p.) was given 30 min earlier. Blood samples were collected at 6 h after decompression. Ani and PNU reduced serum insulin **(A)** and glucose **(C)** and HOMA-IR index **(E)**, and elevated QUICKI **(G)** in C57BL/6 mice after decompression, and MLA significantly counteracted the effects of Ani. Ani reduced serum insulin **(B)** and glucose **(D)** and HOMA-IR **(F)** index, and elevated QUICKI **(H)** in WT mice for α7nAChR after decompression, which was abolished in α7^-/-^ mice. *N* = 6 per group. ^∗^*P* < 0.05, ^∗∗^*P* < 0.01, ^∗∗∗^*P* < 0.001 vs. control. ^#^*P* < 0.05, ^##^*P* < 0.01, ^###^*P* < 0.001 vs. Ani. Power = 1.00, 1.00, 1.00, 0.91, 1.00, 1.00, 1.00, and 1.00 for **(A–H)** respectively.

Serum insulin and glucose levels and HOMA-IR were substantially increased, and QUICKI index was markedly decreased in rats with crush syndrome compared with normal rats. Ani treatment at 30 min before decompression significantly reduced insulin level (-26.5%, *P* < 0.05, **Figure 3A**), but had no effect on glucose level (**Figure 3B**) in rats with crush syndrome, indicating a higher insulin sensitivity in Ani treated rats. Ani reduced HOMA-IR to (-34.6%, *P* < 0.05, **Figure 3C**) and elevated QUICKI index to (+4.8%, *P* < 0.05, **Figure 3D**) of those in control group, supporting that Ani treatment enhances insulin sensitivity. Treatment with Ani significantly enhanced insulin sensitivity in mice with crush syndrome, reflected by reduced serum insulin (*P* < 0.05), glucose (*P* < 0.01), and HOMA-IR (*P* < 0.05), and elevated QUICKI index (*P* < 0.05). PNU had similar effect with Ani. However, MLA attenuated the effect of Ani on insulin sensitivity, reflected by elevated serum insulin (+29.5%, *P* < 0.05, **Figure 4A**), glucose (+95.6%, *P* < 0.001, **Figure 4C**), and HOMA-IR (+69.8%, *P* < 0.01, **Figure 4E**), and reduced QUICKI index (-7.1%, *P* < 0.01, **Figure 4G**). Ani treatment significantly enhanced insulin sensitivity in WT mice, reflected by reduced serum insulin, glucose, and HOMA-IR, and elevated QUICKI index (*P* < 0.05 for all parameters, **Figures 4B,D,F,H**), but had no effect in α7^-/-^ mice.

### Ani Decreases Extracellular K^+^ and Glucose *In Vitro* through Indirectly Activating α7nAChR

**FIGURE 5:**
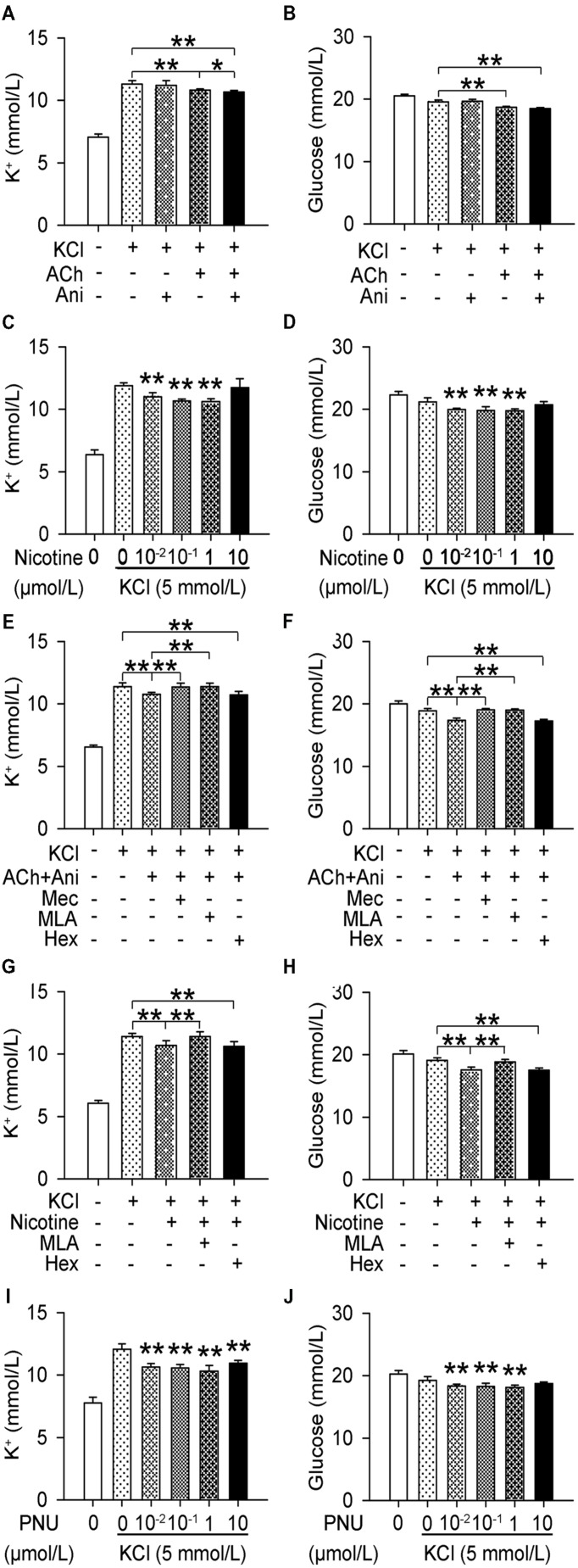
**Involvement of α7nAChR in the effect of Ani on extracellular K^+^ in C2C12 cells.** C2C12 myotubes were pretreated for 1 h with mecamylamine (Mec, 10 μmol/L), MLA (10 μmol/L), or hexamethonium (Hex, 10 μmol/L), and then for 30 min with Ani (500 μg/ml), ACh (100 μmol/L), Ani (500 μg/ml) + ACh (100 μmol/L), nicotine (0.01–10 *μ*mol/L), or PNU282987 (0.01–10 μmol/L) prior to exposure to KCl (5 mmol/L), and extracellular K^+^ and glucose levels were measured 6 h later. Extracellular K^+^ and glucose in cultured myotubes were significantly decreased by Ani + ACh **(A,B)**, nicotine **(C,D)**, and PNU **(I,J)**. Mec and MLA, but not Hex significantly counteracted the effects of Ani + ACh **(E,F)** or nicotine **(G,H)**. *N* = 8 per group. ^∗^*P* < 0.05, ^∗∗^*P* < 0.01. Power = 1.00 for **(A–J)**.

Extracellular K^+^ in cultured myotubes was significantly decreased by ACh (*P* < 0.01), which could be strengthened by Ani (*P* < 0.05, **Figure 5A**), but Ani alone had no effect. Nicotine and PNU decreased extracellular K^+^ in a concentration-dependent manner (**Figures 5C,I**). Pretreatment with mecamylamine (*P* < 0.01) or MLA (*P* < 0.01, **Figure 5E**), but not hexamethonium, attenuated the effect of Ani and ACh on extracellular K^+^. Pretreatment with MLA (*P* < 0.01, **Figure 5G**), but not hexamethonium, attenuated the effect of nicotine on extracellular K^+^. Similar results were obtained for extracellular glucose (**Figures 5B,D,F,H,J**).

### Ani Increases Na/K-ATPase Phosphorylation *In Vitro* Relying on Insulin Receptor

**FIGURE 6:**
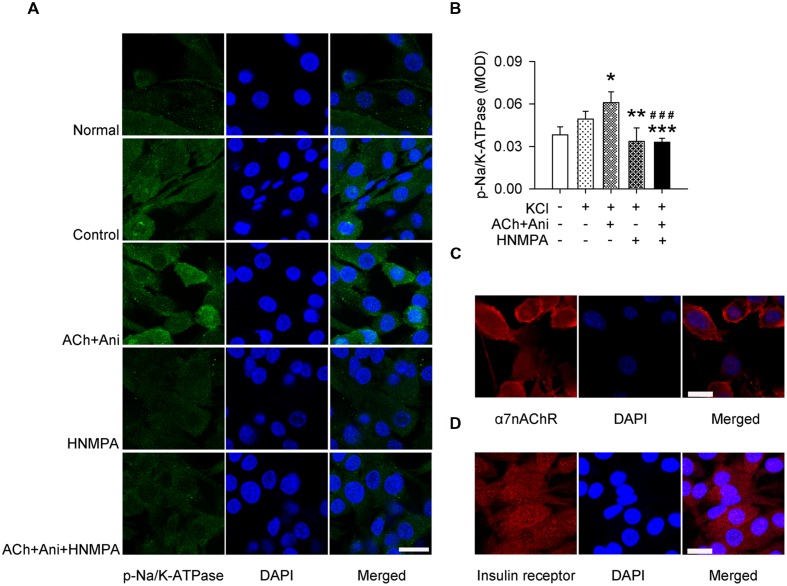
**Influence of HNMPA-(AM)3 (HNMPA) on the effect of Ani on Na/K-ATPase phosphorylation in C2C12 cells.** C2C12 myotubes were pretreated for 1 h with HNMPA (200 μmol/L), and then for 30 min with Ani (500 μg/ml) + ACh (100 μmol/L) prior to exposure to KCl (5 mmol/L). Ani + ACh significantly increased phosphorylation of Na/K-ATPase in cultured myotubes, and HNMPA attenuated such effect (**A,B**, scale bar = 30 μm). Both α7nAChR (**C**, scale bar = 20 μm) and insulin receptor (**D**, scale bar = 30 μm) were expressed in C2C12-differentiated myotubes, detected with immunofluorescence staining and confocal microscopy. *N* = 6 per group. ^∗^*P* < 0.05, ^∗∗^*P* < 0.01, ^∗∗∗^*P* < 0.001 vs. control. ^###^*P* < 0.001 vs. ACh + Ani. Power = 1.00.

Ani and ACh significantly increased phosphorylation of Na/K-ATPase in cultured myotubes (+23.5%, *P* < 0.05). Pretreatment with HNMPA-(AM)3 attenuated the effect of Ani and ACh on Na/K-ATPase phosphorylation (-46.0%, *P* < 0.001) to a level that was significantly lower than control group (-33.3%, **Figures 6A,B**). α7nAChR and insulin receptor were also expressed in C2C12-differentiated myotubes (**Figures 6C,D**).

### Ani Decreases Extracellular K^+^ and Glucose *In Vitro* through Insulin Signaling

**FIGURE 7:**
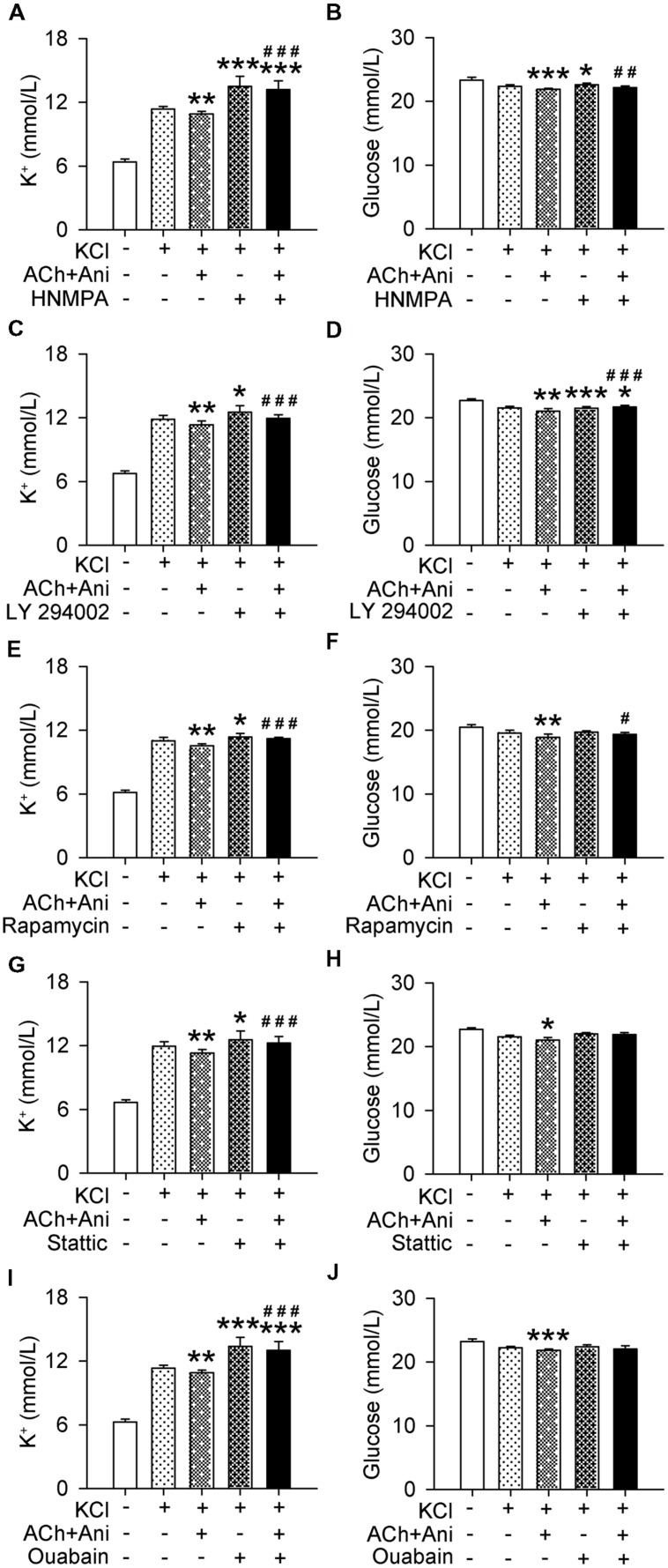
**Influences of insulin signaling blocking on the effect of Ani on extracellular K^+^ in C2C12 cells.** C2C12 myotubes were pretreated for 1 h with HNMPA-(AM)3 (HNMPA, 200 μmol/L), LY 294002 (10 μmol/L), rapamycin (200 nmol/L), stattic (10 μmol/L), or ouabain (2 mmol/L), and then for 30 min with Ani (500 μg/ml) + ACh (100 μmol/L) prior to exposure to KCl (5 mmol/L), and extracellular K^+^ and glucose levels were measured 6 h later. Extracellular K^+^ and glucose in cultured myotubes were significantly decreased by Ani + ACh. HNMPA **(A,B)**, LY 294002 **(C,D)**, and rapamycin **(E,F)** attenuated such effects. Stattic **(G,H)** and ouabain **(I,J)** attenuated the effect of Ani + ACh on extracellular K^+^ but not on glucose. *N* = 8 per group. ^∗^*P* < 0.05, ^∗∗^*P* < 0.01, ^∗∗∗^*P* < 0.001 vs. control. ^#^*P* < 0.05, ^##^*P* < 0.01, ^###^*P* < 0.001 vs. ACh + Ani. Power = 1.00 for **(A–J)**.

Extracellular K^+^ and glucose in cultured myotubes were significantly decreased by Ani and ACh. Pretreatment with HNMPA-(AM)3 or ouabain attenuated the effect of Ani and ACh on extracellular K^+^ (*P* < 0.001 for both drugs) to a level that was significantly higher than control group (+16.2% and +14.9% respectively, **Figures 7A,I**). Pretreatment with LY 294002, rapamycin, or stattic also attenuated the effect of Ani and ACh on extracellular K^+^ (*P* < 0.001 for all drugs, **Figures 7C,E,G**). Pretreatment with HNMPA-(AM)3, LY 294002, or rapamycin, but not stattic or ouabain attenuated the effect of Ani and ACh on extracellular glucose (**Figures 7B,D,F,H,J**).

## Discussion

The major findings of the present study include: (i) activation of α7nAChR with Ani decreases on-site mortality in crush syndrome; (ii) such effect of activating α7nAChR is partially due to decline of serum potassium; (iii) Ani decreases serum potassium through insulin signaling-Na/K-ATPase pathway.

The on-site causes of death in crush syndrome are cardiac arrest caused by hyperkalemia or hypovolemic shock caused by bleeding or fluid redistribution (Ashkenazi et al., 2005). Hemostasis, intravenous fluids supplement, and blood transfusion are required to treat hypovolemic shock, and for hyperkalemia with definite diagnosis, calcium gluconate, insulin with glucose, sodium bicarbonate, or β_2_ adrenergic receptor agonist are recommended to reduce serum potassium quickly, followed by hemodialysis and kayexalate or enema (Gonzalez, 2005; Weisberg, 2008; Sever and Vanholder, 2013). However, there is no safe and effective drug before definite diagnosis in hospital. Ani is widely used clinically in China for treatment of various shocks, especially septic shock with fewer and less severe adverse effects than atropine (Li et al., 1999). In the present study, we provided evidences that Ani could decrease on-site mortality in rats with crush syndrome, especially administrated before decompression. Therefore Ani was given shortly before decompression, KCl injection, or exposure of myotubes to KCl in all of the following experiments. Our previous studies showed that Ani benefited septic shock through cholinergic anti-inflammatory pathway, i.e., indirectly activation of α7nAChR (Liu et al., 2009). Results of this study demonstrated that the beneficial effect of Ani on mortality in crush syndrome was also mediated by activation of α7nAChR, based on the facts that PNU, selective agonist of α7nAChR, has the similar effect to Ani, but α7nAChR deficiency or MLA, selective antagonist of α7nAChR, attenuated such effect of Ani.

Sudden decompression of the crush victims may release toxic cellular components and electrolytes, such as myoglobin, creatinine, and potassium, from necrotic muscles into blood circulation (Santangelo et al., 1982; Noji, 1992). Consistent with these studies, we observed higher serum potassium and more severe muscle destruction and renal dysfunction in rats with crush syndrome. Treatment with Ani could alleviate these symptoms. However, whether alleviation of the above symptoms was the direct cause of decline of mortality was questioned. Inspiringly, we found Ani could decrease both serum potassium and mortality in rats with hyperkalemia by similar degrees to those in crush syndrome. All these results indicated that Ani could decrease on-site mortality in crush syndrome through modest reduction (-10 ∼-20%) of serum potassium with no risk of hypokalemia, although not as markedly as expected. Furthermore, the beneficial effect of Ani on serum potassium was also mediated by activation of α7nAChR in mice with crush syndrome, which indirectly proved the cause-effect relationship between serum potassium and mortality in crush syndrome. Moreover, extracellular potassium and glucose were also proved to be reduced through activating α7nAChR but not other nicotinic ACh receptors (blocked by hexamethonium) in cultured myotubes.

Insulin can decrease serum potassium, accompanying reduction of serum glucose. Enhancement of insulin sensitivity may strengthen such effect on serum potassium. It has been reported that chronic nicotine administration can reduce insulin resistance in obese mice and enhance insulin sensitivity in normal rats via α7nAChR (Wang et al., 2011; Xu et al., 2012). Moreover, α7^-/-^ mice exhibit insulin resistance (Wang et al., 2011; Somm et al., 2014). Our results showed that blood insulin and glucose levels were elevated in rats with crush syndrome, which were reduced by Ani treatment with no risk of hypoglycemia. The dropped insulin sensitivity was also enhanced by Ani, represented as decreased HOMA-IR and increased QUICKI. Furthermore, such effects were also mediated by activation of α7nAChR, proved in mice with PNU, MLA, or α7nAChR deficiency. Na/K-ATPase functions as a solute pump that pumps sodium out of cells while pumping potassium into cells. As reported, insulin increases potassium uptake through activation of Na/K-ATPase (Chibalin et al., 2001; Al-Khalili et al., 2004; Benziane and Chibalin, 2008; Clausen, 2010). In our study, activation of Na/K-ATPase was enhanced by Ani and ACh, but reduced by inhibition of tyrosine kinase on insulin receptor with HNMPA-(AM)3. Besides HNMPA-(AM)3, inhibition of phosphoinositide 3-kinase, mammalian target of rapamycin, signal transducer and activator of transcription 3, and Na/K-ATPase with LY 294002, rapamycin, static, and ouabain respectively could all decrease extracellular potassium. All the results demonstrated that activation of α7nAChR could increase insulin sensitivity to activate downstream signaling and Na/K-ATPase, and to decrease serum potassium consequently.

We also examined the influence of Ani on hypovolemic shock in rats with crush syndrome shortly after decompression. Ani could alleviate hypotension through activation of α7nAChR, but had no effect on heart rate. Such effect was proved in rats with hemorrhagic shock, by elevation of blood pressure and reduction of mortality (data not shown). Therefore, activation of α7nAChR could decrease on-site mortality in crush syndrome through alleviation of both hyperkalemia and hypovolemic shock. The antishock effect of Ani is mediated by cholinergic anti-inflammatory pathway, besides the putative and non-specific mechanisms to improve blood flow in microcirculation (Poupko et al., 2007; Liu et al., 2009). Increasing evidences prove that High mobility group box 1 (HMGB1), a kind of damage-associated molecular pattern molecule, can initiate and perpetuate a non-infectious inflammatory response (Tsung et al., 2005; Rubartelli and Lotze, 2007; Bald et al., 2014; Huang et al., 2015). We found that HMGB1 was dramatically increased in crush syndrome, which could be partially corrected by treatment with Ani. However, neutralization of HMGB1 with its antibody had no influence on the effect of Ani on on-site mortality in mice with crush syndrome (data not shown). The influence of Ani on proinflammatory cytokines in crush syndrome and its relationship with mortality need further investigations.

## Conclusion

**FIGURE 8:**
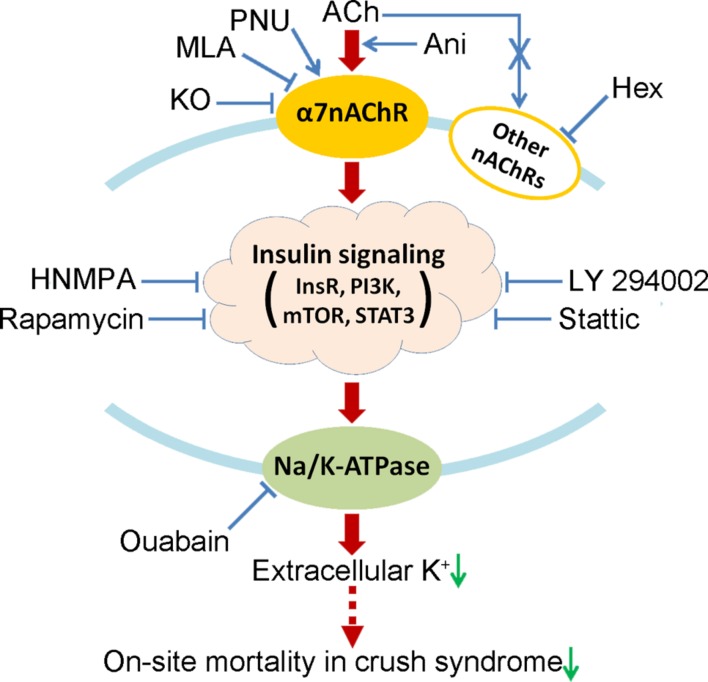
**Proposed mechanism by which activation of α7nAChR decreases on-site mortality in crush syndrome.** Hex, hexamethonium; HNMPA, HNMPA-(AM)3; InsR, insulin receptor; KO, knockout; mTOR, mammalian target of rapamycin; PI3K, phosphoinositide 3-kinase; STAT3, signal transducer and activator of transcription 3.

This study demonstrates that activation of α7nAChR could decrease on-site mortality in crush syndrome based on the decline of serum potassium through insulin signaling-Na/K-ATPase pathway (**Figure 8**). Drugs targeting at activation of α7nAChR, such as Ani, are encouraged for on-site remedy of crush syndrome, which needs further investigations.

### Limitations

In the present study, on-site mortality in crush syndrome was proved to be decreased by activation of α7nAChR through decline of serum potassium. However, changes in the incidence of cardiac arrest caused by hyperkalemia were not detected, which need further investigation. Involvement of insulin sensitivity and Na/K-ATPase in the reduction of on-site mortality in crush syndrome by activation of α7nAChR was not proved by corresponding inhibitors *in vivo*, considering the possibility that the mortality may be increased by disorders in serum glucose and energy supply.

## Author Contributions

J-GY and XL designed the study and experiments; B-SF, E-HZ, MW, and J-MG performed the experiments; J-GY and B-SF analyzed the data; J-GY, B-SF, XL, and D-FS wrote the paper.

## Conflict of Interest Statement

The authors declare that the research was conducted in the absence of any commercial or financial relationships that could be construed as a potential conflict of interest.
